# Supplementary figures and images for: Genome-Wide Identification and Expression Analysis of Auxin Response Factor (ARF) Gene Family in Panax ginseng Indicates Its Possible Roles in Root Development
Source: Plants (Basel). 2023 Nov 23;12(23):3943. doi: 10.3390/plants12233943 (PMC10708364; doi:10.3390/plants12233943)

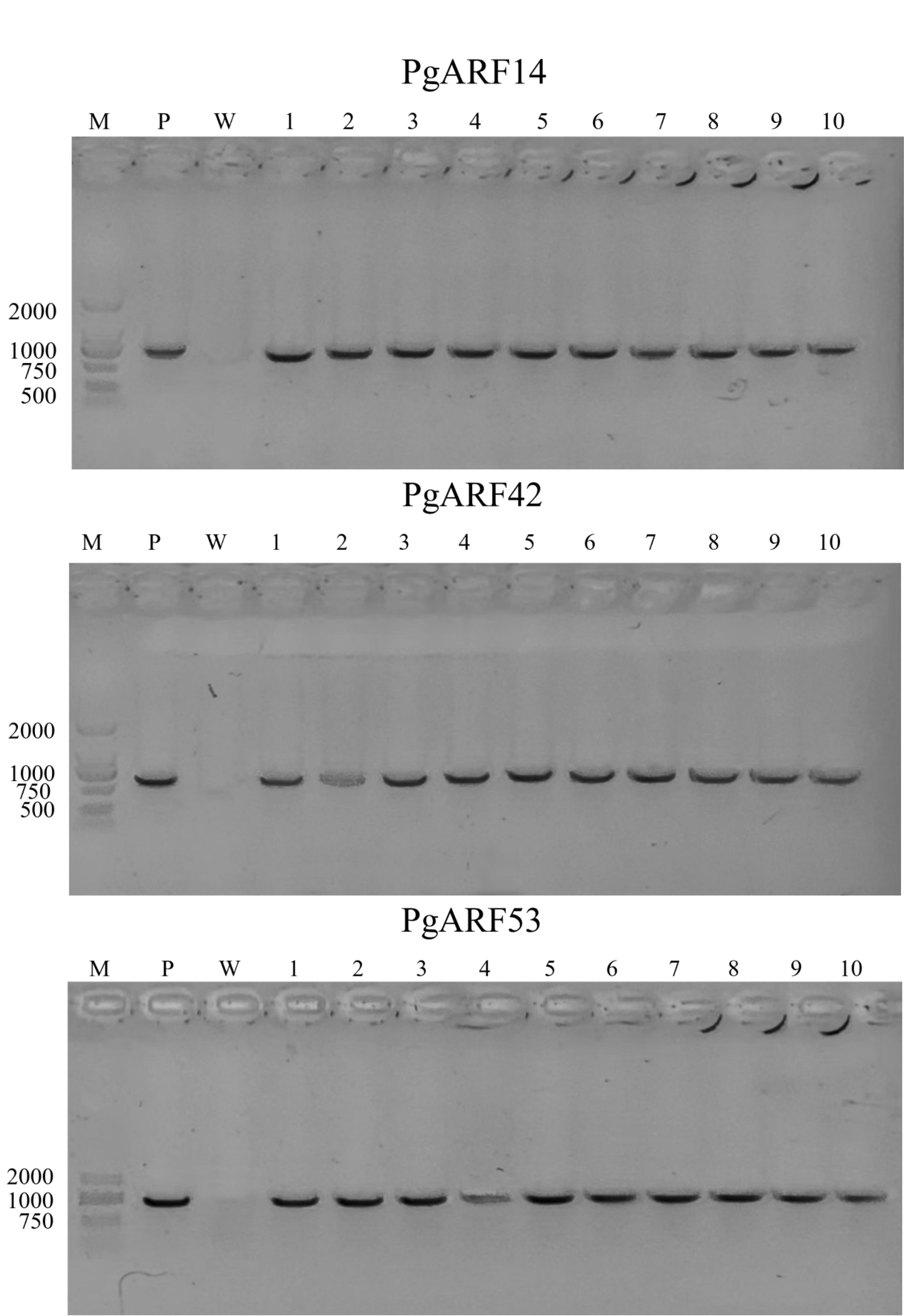

Supplement: Supplementary file 1 [file plants-12-03943-s001.zip › Figure S2. transgenic validation.png]
